# Valorization of Red Grape Pomace Waste Using Polyethylene Glycol and Fibrolytic Enzymes: Physiological and Meat Quality Responses in Broilers

**DOI:** 10.3390/ani9100779

**Published:** 2019-10-10

**Authors:** Cebisa Kumanda, Victor Mlambo, Caven Mguvane Mnisi

**Affiliations:** 1 Department of Animal Science, Faculty of Natural and Agricultural Sciences, North-West University, P Bag x2046, Mmabatho 2735, South Africa; cebisa.kumanda@nwu.ac.za; 2 Food Security and Safety Niche area, Faculty of Natural and Agricultural Sciences, North-West University, P Bag x2046, Mmabatho 2735, South Africa; 3 School of Agricultural Sciences, Faculty of Agriculture and Natural Sciences, University of Mpumalanga, P Bag x11283, Mbombela 1200, South Africa; victor.mlambo@ump.ac.za

**Keywords:** blood parameter, broiler, growth performance, meat quality, polyethylene glycol, red grape pomace waste

## Abstract

**Simple Summary:**

Red grape pomace (GP) waste, although rich in beneficial phenolic compounds, is traditionally disposed in landfills and through incineration, resulting in environmental pollution. The revalorization of GP as a source of nutrients and bioactive compounds in chicken diets is an environmentally sustainable and lower-cost alternative to current disposal methods. This approach has the potential to improve food and nutrition security while providing health benefits to consumers of poultry products. Unfortunately, the amount of GP that can be included in broiler diets is limited by fiber and condensed tannins found in this agro-waste. These compounds reduce the digestibility of GP in chickens, resulting in poor bioavailability of the beneficial bioactive compounds. Strategies are, therefore, required to ameliorate the effects of fiber and condensed tannins. This study investigated whether pre-treating GP with polyethylene glycol (PEG) and a cellulolytic enzyme mixture (Viscozyme^®^) would improve feed intake, physiological parameters, carcass characteristics and meat quality parameters of broilers. It was concluded that PEG treatment successfully ameliorated the anti-nutritional effects of condensed tannins. However, the cellulolytic enzyme treatment was ineffective against GP fiber.

**Abstract:**

The amount of grape pomace (GP) waste that can be included as a functional feed in broiler diets is limited by anti-nutritional compounds such as fiber and condensed tannins. This study evaluated the effect of pre-treating GP with polyethylene glycol (PEG) and a cellulolytic enzyme mixture on physiological and meat quality parameters of broilers. Cobb 500 broilers (249.2 ± 20.31 g live-weight) were reared on five isoenergetic and isonitrogenous diets: 1. Commercial chicken diet (CON); 2. CON containing untreated GP at 100 g/kg (dGP); 3. CON containing 100 g/kg GP pre-treated with PEG (50 g/kg) (dPEG); 4. CON containing 100 g/kg GP pre-treated with enzyme (1 g/kg) (ENZ); and 5. CON containing 100 g/kg GP pre-treated with PEG (50 g/kg) and enzyme (1 g/kg) (PENZ). Overall body weight gains were similar in broilers reared on the CON, dPEG, ENZ and PENZ diets but lower in dGP chickens. The meat of birds reared on dPEG, ENZ, dGP and CON had a similar water-holding capacity, which was lower than in PENZ chickens. Diets influenced the size of duodenum, ileum, jejunum and caeca. Polyethylene glycol treatment promoted similar body weight gains and hot carcass weights as the commercial control diet, suggesting that the anti-nutritional effects of condensed tannins were successfully ameliorated.

## 1. Introduction

The traditional approach of disposing large volumes of red grape pomace waste (GP) in landfills and through incineration poses a major environmental challenge [1]. The valorization of GP could help maintain environmental equilibrium through waste reduction while providing additional economic benefits. Using this agro-waste as a functional feedstuff in broiler diets is a re-valorization and waste reduction strategy with the potential to improve food and nutrition security and environmental stewardship. Indeed, the alternative use of GP as a source of potentially beneficial phytochemical compounds in broiler diets has been evaluated with promising results [2,3]. These phytochemical compounds are reported to have antioxidative, antimicrobial and health-promoting effects [4,5]. Consequently, GP is a potential functional feedstuff for broilers with polyphenols that has been demonstrated to enhance the oxidative stability of meat and to promote the proliferation of beneficial intestinal bacteria [2,6]. However, the bioavailability of beneficial bioactive compounds in this by-product is rather low due to high fiber (43–75%) [7] and condensed tannin (20–30%) [8] content. Both condensed tannins and fiber are known to negatively affect the digestibility of diets [9], especially in simple non-ruminants such as chickens [10]. Azizi et al. [6] reported that high amounts of tannins in GP reduce feed intake and negatively affect feed efficiency in chickens. This, in turn, limits the amount of GP that could be included in broiler diets to the detriment of red grape pomace re-valorization and waste reduction efforts.

Strategies to counter the anti-nutritional effects of condensed tannins and fiber need to be identified and evaluated in order to improve the bioavailability of bioactive compounds from GP. Pre-treatment of GP with exogenous fibrolytic enzymes and/or polyethylene glycol (PEG) may allow for higher dietary inclusion levels of GP in chicken diets. While the utility of cell wall-degrading feed enzymes in improving the feed value of feed ingredients is generally well documented, their use in enhancing the utilization of GP in birds is limited to pectinases and cellulases [11]. On the other hand, PEG is known for its ability to bind tannins, thus freeing proteins and other dietary components for digestion and absorption [12]. Indeed, Besharati and Taghizadeh [13] reported that PEG breaks already formed tannin–protein complexes, due to its high tannin affinity. This was also confirmed by Hlatini et al. [14] when PEG was included in *Acacia tortilis* leaf meal diets for pigs. While the use of PEG to inactivate condensed tannins in ruminant diets has been widely investigated, there are no documented studies on its application to improve GP utilization in broiler chickens. The possible effects of inactivating condensed tannins using PEG on physiological and meat quality parameters of broilers are also unknown. In addition, this study is the first to explore possible additive effects of PEG and cell wall-degrading enzymes as a strategy to valorize red grape pomace waste for broilers. Therefore, this study was designed to determine the effect of PEG and Viscozyme^®^ treatment of dietary GP on growth, blood parameters, and carcass and meat quality traits of broilers. The research hypothesis that pre-treating GP with fibrolytic enzymes and/or PEG will improve physiological parameters and meat quality traits of Cobb 500 broiler chickens was tested.

## 2. Materials and Methods

Rearing and slaughter procedures of the research birds were approved by the Animal Research Ethics Committee of the North-West University (approval no. NWU-00239-18-A5), which conforms to the guidelines and use of research animals.

### 2.1. Study Site and Ingredient Sources

Red grape (*Vitis vinifera L.* var. Shiraz) pomace (GP) was sourced from Blaauwklippen Wine Estate (33.969° S; 18.844° E) (Stellenbosch, South Africa), the soil types of which ranged from dark alluvial to clay. In this Estate, daily temperature averages 16.4 °C, while annual rainfall averages 802 mm. Associated Chemical Enterprises (Johannesburg, South Africa) supplied PEG (Mr 4000), while the enzyme Viscozyme^®^ L (a cellulolytic mixture of arabinase, cellulase, β-glucanase, hemicellulase and xylanase enzymes, with an equivalent enzymatic activity of 100 fungal beta-glucanase per gram), were supplied by Sigma–Aldrich, Modderfontein, South Africa. The broiler feeding trial was conducted at Molelwane Research Farm (33.969° S; 18.844° E) during the winter season, when ambient temperatures ranged from −3 to 25 °C. The broiler house was fitted with semi-automatic curtains that were rolled down in the morning and closed in the evening. The temperature in the pens was monitored using a thermometer and light was supplied using fluorescent lights. Sunflower husks were used as bedding in all the pens.

### 2.2. Pre-Treatment of Red Grape Pomace with Polyethylene Glycol and Enzyme

Red grape pomace (5 kg per treatment) was pre-treated with aqueous solutions of PEG (5 g PEG/100 g milled GP), Viscozyme^®^ (0.1 g enzyme/100 g milled GP), and a combination of PEG and enzyme. For the PEG treatment, 5 kg GP was sprayed and mixed with 5 L of distilled water in which 250 g of PEG had been dissolved. For the enzyme treatment, 5 kg of GP was sprayed and mixed with 5 L of distilled water in which 4.2 mL of Viscozyme^®^ (density: 1.2 g/mL) had been added. For the combined treatment of PEG and Viscozyme^®^, 250 g of PEG and 4.2 mL of Viscozyme^®^ were both dissolved in 5 L of distilled water, which was then sprayed on 5 kg GP. The untreated GP (5 kg) was sprayed with 5 L of distilled water only. The amount of distilled water used to dissolve both the PEG and enzyme was determined through an iterative process, with the objective of avoiding excess run-off liquid that would have leached the GP. Treated and untreated GP were stored for a period of 24 h under room temperature to allow time for PEG and Viscozyme^®^ to react with GP tannins and fiber, respectively. At the end of this incubation period, treated and untreated GP were oven-dried at 50 °C and then crushed to break up lumps before being incorporated into commercial grower and finishing diets.

### 2.3. Diet Formulation

Five isonitrogenous and isoenergetic experimental diets were formulated to meet the daily nutritional requirements of growing and finishing chickens according to National Research Council [15] guidelines. The diets, for grower and finisher phases, were formulated by including treated or untreated GP at 100 g/kg as follows: (1) Commercial chicken diet without red grape pomace (CON); (2) Commercial chicken diet containing 100 g/kg untreated red grape pomace (dGP); (3) Commercial chicken diet containing 100 g/kg red grape pomace pre-treated with PEG (50 g/kg) (dPEG); (4) Commercial chicken diet containing 100 g/kg red grape pomace pre-treated with Viscozyme^®^ (1 g/kg) (ENZ); and (5) Commercial chicken diet containing 100 g/kg GP pre-treated with PEG (50 g/kg) and Viscozyme^®^ (1 g/kg) (PENZ). The ingredient composition of the five diets is presented in Table 1.

### 2.4. Chemical Analyses

Samples of diets were milled and analyzed using the Official Analytical Chemists International methods [16]: 930.15 for dry matter, 924.05 for organic matter, 984.13 for crude protein, 978.10 for crude fiber and 920.39 for crude fat (Table 2). Near infrared reflectance spectroscopy models were used to predict the metabolizable energy content of the diets. Minerals were analyzed following the Agricultural Laboratory Association of Southern Africa [17] guidelines.

### 2.5. Feeding Trial

Four hundred, day-old mixed-gender Cobb 500 broiler chicks (OptiChicks, South Africa) were fed a starter mash diet supplied by Nutri-Feeds (South Africa) for a period of 10 days. The chicks were allocated to 40 pens (3.5 m long × 1.0 m wide × 1.85 m high), with each pen (experimental unit) carrying 10 birds. The five experimental diets were randomly allocated to the pens (8 replicates per diet). Days 11 to 13 were used as an adaptation period such that measurements were only taken from day 14 to day 42. Clean fresh water was provided at all times and rearing was done under natural lighting (10 h of daylight).

### 2.6. Feed Intake, Growth Performance and Blood Analyses

Average weekly feed intake (AWFI), average weekly body weight gain (ABWG) and the feed conversion ratio (FCR) were determined as described by Kumanda et al. [2]. At 40 days of age, two broiler chickens, randomly selected from each pen, were used to collect blood from the brachial vein. Whole blood and sera blood were analyzed using an automated IDEXX LaserCyte Haematology Analyser and an automated IDEXX Catalyst One Chemistry Analyser (IDEXX Laboratories Inc., Maine, US), respectively.

### 2.7. Carcass Characteristics and the Size of Internal Organs

On day 42, feed was withheld for 13 h before the birds were weighed (slaughter body weight) and transported to an abattoir. All 10 birds from each replicate pen were electrically stunned, slaughtered, bled and defeathered. Carcass weights (Explorer EX224, 0.01 g readability (2 decimal places), supplied by OHAUS Corp, Parsippany, NJ, USA) were taken immediately after slaughter (hot carcass weight (HCW)) and also after chilling at 4 °C for 24 h (cold carcass weight, CCW). The dressing out percentage was determined as a proportion of HCW to slaughter body weight. The size of internal organs (liver, gizzard, heart, proventriculus, spleen, pancreas, duodenum, ileum, jejunum, large intestine, caeca and lungs) were measured and expressed as a proportion of HCW.

### 2.8. Meat Quality Traits

All carcasses from each replicate pen and treatment were used to measure meat quality parameters. Breast meat pH and temperature were recorded 24 h post-mortem using a Corning Model 4 pH/temperature meter (Corning Glass Works, Medfield, MA). Meat color indices (L * = lightness, a * = redness and b * = yellowness) were determined on the inner surface of raw thigh muscle, 24 h post-mortem using a Minolta color guide (BYK-Gardener GmbH, Geretsried, Germany) according to Commission Internationale de l’Eclairage [18]. Hue angle and chroma were calculated according to Priolo et al. [19]. The water-holding capacity (WHC) of the pectoralis major muscle (8–16 g) was determined as described by Mulaudzi et al. [20]. Drip loss [21] and cooking loss [22] were determined using breast meat samples. The samples used for cooking loss determination were then used for shear force determination with a Texture Analyser (TA XT plus, Stable Micro Systems, Surrey, UK).

### 2.9. Statistical Analysis

Data for each parameter collected per replicate pen were averaged first before statistical analysis. The NORMAL option in the Proc Univariate statement was used to test for normality of measured parameters. Repeated measures procedures of Statistical Analysis System (SAS) [23] were used to analyze data for parameters measured weekly (feed intake, body weight gain and feed conversion ratio). Overall growth performance parameters as well as blood parameters, carcass characteristics and meat quality data were analyzed using the general linear model procedure of SAS [23] according to the following linear statistical model:

Y_ik_ = μ + D_i_ + E_ik_ (1)

where Y_ik_ = response variable, *µ* = overall mean, D_i_ = dietary effect and E_ik_ = random error associated with observation ik, assumed to be normally and independently distributed. For all statistical tests, significance was declared at *p* < 0.05.

## 3. Results

### 3.1. Feed Intake, Growth Performance and Blood Parameters

There were no significant (*p* > 0.05) week × diet interaction effects on AWFI, ABWG and FCR. Table 3 shows that there were no significant (*p* > 0.05) dietary influences on overall feed intake (g/bird), initial body weight (g/bird) and overall FCR. The CON diet promoted the highest overall BWG (1351.4 g/bird) and final BW (1653.2 g/bird), which did not differ (*p* > 0.05) from dPEG, ENZ and PENZ diets. However, birds on untreated GP had the lowest body weight gain (1188.9 g/bird) and final BW (1468.4 g/bird).

With the exception of mean corpuscular volume (MCV), all hematological parameters were not (*p* > 0.05) influenced by dietary treatments (Table 4). Broilers fed ENZ had a higher MCV (34.87 fL) when compared to those on CON (27. 66 fL), dGP (27.70 fL) and dPEG (27.89 fL) diets.

With the exception of phosphorus, all serum biochemical parameters were not significantly affected by dietary treatments (Table 5). Broilers on dGP had the lowest serum phosphorus level, which did not differ (*p* > 0.05) with those on CON and PENZ diets. Broilers on ENZ had the highest serum phosphorus level (4.74 mmol/L), which did not differ from dPEG and CON diets.

### 3.2. Carcass Characteristics, Internal Organs and Meat Quality Parameters

Table 6 shows that there were significant dietary effects on slaughter BW, HCW and CCW of broiler chickens (*p* < 0.05). The slaughter body weights of CON, dPEG, ENZ and PENZ chickens did not differ (*p* > 0.05). However, dGP diet promoted the lowest slaughter body weight (1468.4 g) in chickens. Broiler chickens on CON (1276.5 g) and dPEG (1243.6 g) diets had a higher HCW, which did not differ. However, dGP promoted the lowest (1120.6 g) HCW while the HCW of dPEG, ENZ and PENZ chickens did not differ (*p* > 0.05). Broilers on the CON (1227.4 g) and dPEG (1210.0 g) diets had a higher CCW compared to those fed dGP, ENZ and PENZ diets, whose CCW did not differ.

There were no dietary effects (*p* > 0.05) on the size of the liver (2.2–2.3 g/100 g HCW), gizzard (2.3–2.5 g/100 g HCW), heart (0.6–0.7 g/100 g HCW), proventriculus (0.5–0.6 g/100 g HCW), spleen (0.1–0.2 g/100 g HCW), pancreas (0.2–0.3 g/100 g HCW), large intestine (0.2–0.7 g/100 g HCW) and lung (0.6–0.7 g/100 g HCW) of broiler chickens. However, broilers on the CON diet had the lightest duodenum (0.7 g/100 g HCW), while those on dGP, dPEG ENZ and PENZ diets had the heaviest duodenum. Chickens on the CON diet had the lightest ileum (1.4 g/100 g HCW), which did not differ from those fed dPEG, ENZ and PENZ diets. Birds on the dGP diet had the heaviest ileum (1.7 g/100 g HCW), which did not differ from those fed dPEG, ENZ and PENZ diets. The CON experimental diet promoted a lighter (*p* < 0.05) jejunum (1.4 g/100 g HCW) compared to dGP, dPEG, ENZ and PENZ diets, which did not differ. The broilers on CON (0.8 g/100 g HCW) and dPEG (1.0 g/100 g HCW) diets had the lightest caeca, while birds on dGP and PENZ diets had the heaviest caeca.

Table 7 shows that diets influenced the WHC of breast meat with PENZ promoting the highest WHC (8.32%) and dGP promoting the lowest (5.22%). Experimental diets had no effect on meat temperature 24 h after slaughter, meat pH, *L* *, *a* *, *b* *, chroma and hue angle of broiler chickens.

## 4. Discussion

Red grape pomace contains high levels of fiber and polymeric polyphenols such as proanthocyanidins that reduce the digestion and absorption of nutrients and other dietary compounds. As such, the incorporation of high levels of GP in chicken diets might impair nutrient digestion and growth. The application of PEG to ameliorate the anti-nutritional effects of condensed tannins in animal diets has been widely practiced; however, there are no documented studies of its application to improve the utilization of dietary GP in broiler chickens. In addition, possible additive effects of PEG and cell wall-degrading enzymes on physiological and meat quality parameters of broiler chickens have not been investigated. This is despite the possibility that a combined treatment of PEG and Viscozyme^®^ may allow for higher inclusion levels of GP in broiler diets, resulting in reduced feed costs, improved broiler meat quality and good environmental stewardship.

Results from this study suggest that the inclusion of PEG and/or fibrolytic enzyme-treated GP in chicken diets at 100 g/kg did not depress overall feed intake. Indeed, the feed intake in chickens receiving treated GP was similar to that of birds on the commercial control. These results are in agreement with Chamorro et al. [24], who found no effect of carbohydrases and tannase enzymes on the feed intake of male broiler Cobb chicks when reared on diets containing GP. In this study, it was expected that broilers fed a commercial diet containing GP pre-treated with a combination of PEG and Viscozyme^®^ (PENZ) would have the highest overall feed intake. This is because Viscozyme^®^ has the capacity to hydrolyze complex plant cell walls, while PEG binds to tannins, thereby neutralizing their anti-nutritional effects. It was, therefore, anticipated that GP intake would be enhanced through the additive effects of these two treatments. However, the results did not support this hypothesis and no differences in feed intake were observed across all the experimental diets.

The application of PEG and Viscozyme^®^ to GP affected the overall body weight gain of broiler chickens. The pre-treated GP promoted similar overall WG as the commercial control diet, which was higher than for untreated GP, suggesting that the anti-nutritional effects of tannins and fiber were successfully ameliorated, leading to enhanced nutrient bioavailability. These findings contradict those of Chamorro et al. [11], who investigated the effect of an exogenous enzyme, tannase, on the utilization of polyphenolics as well as the oxidation of meat lipids in chicks fed GP. This could be because Chamorro et al. [11] only used cell wall-degrading enzymes but did not attempt to ameliorate the negative nutritional effects of condensed tannins. However, a recent study by Ebrahimzadeh et al. [25] showed that treating GP (included at 100 g/kg level) with a tannin-degrading enzyme, tannase, did not affect the chick growth performance. The fact that PEG treatment improved the body weight gain of chickens in this study shows that this tannin-binding compound may be a better alternative to the enzyme tannase as a strategy to reduce the anti-nutritional effects of tannins. The effect of treated GP-containing diets on growth performance is an indication that PEG and Viscozyme^®^ treatment increased the amount of nutrients released in the intestines. Generally, high tannin diets reduce growth performance as shown by the reduction in the overall WG of broilers reared on the commercial diet containing untreated GP. 

In a study conducted by Lichovnikova et al. [26], feeding broiler chickens with a 1.5% diet of red GP had a positive effect on the antioxidant activity of the blood, indicating its potential as a biological antioxidant. In this study, most of the hematological and serum biochemical parameters were not influenced by dietary treatments and fell within the normal range for chickens. Nonetheless, mean corpuscular volume (MCV), an anemia diagnostic parameter [27] and phosphorus levels were higher on Viscozyme^®^-treated diets indicating that treating GP with Viscozyme^®^ did not compromise the health status of the birds. In contrast to the current findings, Aditya et al. [28] reported lower serum total cholesterol levels in broilers fed GP. However, Kara et al. [29] reported no change in serum triglyceride and total cholesterol levels when laying hens were supplemented with GP.

Theoretically, the consumption of high fiber diets is expected to induce changes in the size of intestines and gizzards in birds as an adaptation mechanism [30]. However, the relative weights of livers, intestines, gizzards and pancreas were not affected by the diets. Although these results were not expected, they are in agreement with Brenes et al. [31] who reported that broilers fed diets containing GP showed no significant differences in the relative weight of internal organs when compared with the control groups. The expectation was that untreated and enzyme-treated GP would still contain phenolics that require detoxification by the liver upon absorption from the digestive tract, leading to atrophy of the organ. However, it is quite possible that at a 100 g/kg inclusion rate, GP did not supply enough phenolics to overwhelm the chicken’s detoxifying mechanisms. For untreated and PEG-treated GP, no reduction in fiber levels was expected, which should have resulted in an increase in the size of the gizzard compared to the control diet.

Chickens reared on the diet containing untreated GP had a lower slaughter body weight compared to those on diets containing treated GP. Treating GP with PEG and Viscozyme^®^ resulted in bigger carcasses due to amelioration of the anti-nutritional effects of tannins and fiber. However, these findings contradict those of Brenes et al. [31], who reported that graded levels of GP and vitamin E had no effect on carcass weight in chickens. The lack of dietary influence on meat color in this study was surprising given that GP contains anthocyanins that cause meat pigmentation. Indeed, a study by Kasapidou et al. [32] on the effect of grape pomace supplementation on broiler meat quality characteristics confirms that dietary GP affects meat redness.

## 5. Conclusions

The dietary inclusion of untreated red grape pomace at 100 g/kg reduced body weight gain in broilers. However, when red grape pomace was pre-treated with polyethylene glycol, broilers had a similar body weight gain and hot carcass weight as those on the commercial control diet. It was concluded that enzyme and polyethylene glycol pre-treatments promoted a similar body weight gain as the conventional commercial diet, suggesting that the anti-nutritional effects of tannins and fiber were successfully ameliorated.

## Figures and Tables

**Table 1:** Ingredient composition (g/kg dry matter, unless otherwise stated) of the experimental diets.

Ingredients	Diets ^1^									
	Grower					Finisher				
	CON	dGP	dPEG	ENZ	PENZ	CON	dGP	dPEG	ENZ	PENZ
Polyethylene glycol	0	0	5	0	5	0	0	5	0	5
Viscozyme^®^-L	0	0	0	0.1	0.1	0	0	0	0.1	0.1
Grape pomace	0	100	100	100	100	0	100	100	100	100
Soy oilcake	245	12	12	12	12	168	0	0	0	0
Fullfat soya	10	229	229	229	229	55	262	262	262	262
Gluten 60	5	38	38	38	38	0	0	0	0	0
Sint lysine	1.39	2.71	2.71	2.71	2.71	1.93	1.52	1.52	1.52	1.52
Methionine	1.42	0.8	0.8	0.8	0.8	1.51	0.97	0.97	0.97	0.97
Threonine	0	0.01	0.01	0.01	0.01	0.1	0	0	0	0
Yellow maize	709	589	589	589	589	751	601	601	601	601
Feed lime	14.6	13	13	13	13	12.5	11.3	11.3	11.3	11.3
Monocalcium phosphate	7	7.9	7.9	7.9	7.9	2.2	2.2	2.2	2.2	2.2
Salt—fine	3.29	3.35	3.35	3.35	3.35	2.78	3.11	3.11	3.11	3.11
Sodium bicarbonate	1.59	1.45	1.45	1.45	1.45	1.91	1.28	1.28	1.28	1.28
Axtra phytase	0.1	0.1	0.1	0.1	0.1	0.1	0.1	0.1	0.1	0.1
Choline	0.8	0.8	0.8	0.8	0.8	0	0	0	0	0
Salinomycin	0.5	0.5	0.5	0.5	0.5	0	0	0	0	0
Olaquindox	0.4	0.4	0.4	0.4	0.4	0.2	0.2	0.2	0.2	0.2
Premix	0.5	0.5	0.5	0.5	0.5	2.5	2.5	2.5	2.5	2.5
Zinc bacitracin	0	0	0	0	0	0.5	0.5	0.5	0.5	0.5
Oil crude soya	0	0	0	0	0	0	13.17	13.17	13.17	13.17

^1^ Diets: CON = commercial chicken diet without red grape pomace; dGP = commercial chicken diet containing 100 g/kg untreated red grape pomace; dPEG = commercial chicken diet containing 100 g/kg red grape pomace pre-treated with polyethylene glycol (50 g/kg); ENZ = commercial chicken diet containing 100 g/kg red grape pomace pre-treated with Viscozyme^®^-L (1 g/kg); PENZ = commercial chicken diet containing 100 g/kg red grape pomace pre-treated with polyethylene glycol (50 g/kg) and Viscozyme^®^-L (1 g/kg).

**Table 2:** Chemical composition (g/kg dry matter, unless otherwise stated) of the experimental diets.

Parameters	Diets ^1^									
	Grower					Finisher				
	CON	dGP	dPEG	ENZ	PENZ	CON	dGP	dPEG	ENZ	PENZ
Dry matter (g/kg)	893.7	906.7	906.7	906.7	906.7	888.6	904.2	904.2	904.2	904.2
^2^ ME (MJ/kg)	11.9	11.9	11.9	11.9	11.9	12.2	12.2	12.2	12.2	12.2
Crude protein	177.1	170.1	170.1	170.1	170.1	160.0	160.0	160.0	160.0	160.0
Crude fat	33.5	72.6	72.6	72.6	72.6	42.65	89.8	89.8	89.8	89.8
Crude fiber	25.0	74.6	74.6	74.6	74.6	35.2	80.7	80.7	80.7	80.7
Organic matter	844.1	857.3	857.3	857.3	857.3	849	862.5	862.5	862.5	862.5
Calcium	8.2	8.2	8.2	8.2	8.2	6.5	6.5	6.5	6.5	6.5
Phosphorus	5.0	4.7	4.7	4.7	4.7	3.4	3.28	3.28	3.28	3.28
Chloride	2.8	3.0	3.0	3.0	3.0	2.5	2.5	2.5	2.5	2.5

^1^ Diets: CON = commercial chicken diet without red grape pomace; dGP = commercial chicken diet containing 100 g/kg untreated red grape pomace; dPEG = commercial chicken diet containing 100 g/kg red grape pomace pre-treated with polyethylene glycol (50 g/kg); ENZ = commercial chicken diet containing 100 g/kg red grape pomace pre-treated with Viscozyme^®^-L (1 g/kg); PENZ = commercial chicken diet containing 100 g/kg red grape pomace pre-treated with polyethylene glycol (50 g/kg) and Viscozyme^®^-L (1 g/kg). ^2^ ME = metabolizable energy.

**Table 3:** Effect of treating red grape pomace with polyethylene glycol and a cellulolytic enzyme mixture on overall feed intake (14–42 d), initial body weight (14 d), final body weight (42 d), and overall body weight gain (14–42 d) and the overall feed conversion ratio (14–42 d) of broiler chickens.

^2^ Parameters	Diets ^1^					^3^ SEM	*p* Value
	CON	dGP	dPEG	ENZ	PENZ		
Overall FI (g/bird)	2957.5	2844.9	2931.6	2930.8	2913.6	40.52	0.371
Initial BW (g/bird)	301.8	279.4	295.5	288.0	293.6	7.13	0.255
Final BW (g/bird)	1653.2 ^b^	1468.4 ^a^	1604.4 ^a,b^	1523.0 ^a,b^	1564.9 ^a,b^	35.53	0.009
Overall BWG (g/bird)	1351.4 ^b^	1188.9 ^a^	1308.9 ^a,b^	1234.9 ^a,b^	1271.3 ^a,b^	35.69	0.027
^3^ Overall FCR	2.20	2.40	2.24	2.24	2.29	0.061	0.057

^1^ Diets: CON = commercial chicken diet without red grape pomace; dGP = commercial chicken diet containing 100 g/kg untreated red grape pomace; dPEG = commercial chicken diet containing 100 g/kg red grape pomace pre-treated with polyethylene glycol (50 g/kg); ENZ = commercial chicken diet containing 100 g/kg red grape pomace pre-treated with Viscozyme^®^-L (1 g/kg); PENZ = commercial chicken diet containing 100 g/kg red grape pomace pre-treated with polyethylene glycol (50 g/kg) and Viscozyme^®^-L (1 g/kg). ^2^ Parameters: Overall FI = feed intake from 14 to 42 days of age; Initial BW = initial body weight at 14 days of age; Final BW = final body weight at 42 days of age; Overall WG = body weight gain from 14 to 42 days of age; Overall FCR = feed conversion ratio from 14 to 42 days of age; ^3^ SEM: Standard error of the mean. ^a,b^ In a row, dietary treatment means with common superscripts do not differ (*p* > 0.05).

**Table 4:** Effect of treating red grape pomace with polyethylene glycol and cellulolytic enzyme on hematological parameters of broiler chickens.

^2^ Parameters	^1^ Diets					^3^ SEM	*p* Value
	CON	dGP	dPEG	ENZ	PENZ		
Erythrocytes (×10^12^/L)	1.26	1.47	1.33	1.05	1.23	0.309	0.909
Hematocrits (L/L)	5.48	5.73	5.41	6.19	5.47	0.565	0.859
Hemoglobin (g/dL)	9.41	9.89	10.08	9.74	9.52	0.267	0.413
MCV (fL)	27.66 ^a^	27.70 ^a^	27.89 ^a,b^	34.87^c^	33.48^bc^	1.432	0.001
MCH (pg)	49.17	51.98	53.31	52.52	59.09	6.195	0.846
RDW (×10^9^/L)	40.81	40.69	39.58	38.74	36.73	1.106	0.078
Reticulocytes (K/µL)	235.2	186.1	178.3	86.0	86.2	50.87	0.176
Lymphocytes (×10^9^/L)	19.85	27.56	93.92	52.11	50.71	26.141	0.277
Neutrophils (×10^9^/L)	3.54	3.97	3.73	4.15	4.56	0.494	0.640
Monocytes (×10^9^/L)	1.91	2.01	2.36	2.48	1.85	0.356	0.649
Eosinophils (×10^9^/L)	0.55	0.66	0.82	0.84	0.79	0.134	0.508
Basophils (×10^9^/L)	0.11	0.12	0.14	0.16	0.16	0.020	0.294

^1^ Diets: CON = commercial chicken diet without red grape pomace; dGP = commercial chicken diet containing 100 g/kg untreated red grape pomace; dPEG = commercial chicken diet containing 100 g/kg red grape pomace pre-treated with polyethylene glycol (50 g/kg); ENZ = commercial chicken diet containing 100 g/kg red grape pomace pre-treated with Viscozyme^®^-L (1 g/kg); PENZ = commercial chicken diet containing 100 g/kg red grape pomace pre-treated with polyethylene glycol (50 g/kg) and Viscozyme^®^-L (1 g/kg). ^2^ Parameters: MCV = mean corpuscular volume; MCH = mean corpuscular haemoglobin; RDW = red cell distribution width; ^3^ SEM: Standard error of the mean. ^a,b,c^ In a row, dietary treatment means with common superscripts do not differ (*p* > 0.05).

**Table 5:** Effect of treating red grape pomace with polyethylene glycol and a cellulolytic enzyme mixture on serum biochemical parameters of broiler chickens.

^2^ Parameters	^1^ Diets					^3^ SEM	*p* Value
	CON	dGP	dPEG	ENZ	PENZ		
Glucose (mmol/L)	8.06	6.70	8.54	8.54	8.06	1.241	0.827
Creatinine (µmol/L)	13.81	10.94	18.00	15.75	15.38	2.416	0.340
Urea (mmol/L)	0.66	0.66	0.71	0.69	0.70	0.021	0.354
Phosphorus (mmol/L)	3.88 ^a,b^	3.30 ^a^	4.54 ^a,b^	4.74 ^b^	3.64 ^a,b^	0.300	0.008
Calcium (mmol/L)	2.24	2.18	2.20	2.55	2.56	0.241	0.644
Total protein (g/L)	51.38	51.88	55.11	59.38	62.06	3.776	0.215
Albumin (g/L)	19.25	18.88	21.38	23.88	22.19	1.990	0.375
Globulin (g/L)	33.44	33.13	31.50	35.44	39.31	2.465	0.228
ALT (U/L)	53.81	55.13	50.00	71.50	64.44	6.913	0.192
ALKP (U/L)	696.1	508.1	566.7	640.6	676.9	94.42	0.600
GGT (U/L)	17.06	15.81	18.88	15.75	12.94	1.762	0.219
Total bilirubin (µmol/L)	11.81	15.38	14.19	20.94	18.50	2.870	0.204
Amylase (U/L)	547.9	461.4	472.3	564.3	516.6	42.81	0.364
Lipase (U/L)	317.3	298.6	371.3	465.3	445.6	60.82	0.223
Cholesterol (mmol/L)	6.24	6.14	6.04	6.57	6.68	0.450	0.818

^1^ Diets: CON = commercial chicken diet without red grape pomace; dGP = commercial chicken diet containing 100 g/kg untreated red grape pomace; dPEG = commercial chicken diet containing 100 g/kg red grape pomace pre-treated with polyethylene glycol (50 g/kg); ENZ = commercial chicken diet containing 100 g/kg red grape pomace pre-treated with Viscozyme^®^-L (1 g/kg); PENZ = commercial chicken diet containing 100 g/kg red grape pomace pre-treated with polyethylene glycol (50 g/kg) and Viscozyme^®^-L (1 g/kg). ^2^ Parameters: ALT = alanine transaminase; ALKP = alkaline phosphate; GGT = gamma glutamyl transferase; ^3^ SEM: Standard error of the mean. ^a,b^ In a row, dietary treatment means with common superscripts do not differ (*p* > 0.05).

**Table 6:** Effect of treating dietary red grape pomace with polyethylene glycol and a cellulolytic enzyme mixture on internal organs and carcass characteristics (g/100 g HCW, unless otherwise stated) of broiler chickens.

^2^ Parameters	^1^ Diets					^3^ SEM	*p* Value
	CON	dGP	dPEG	ENZ	PENZ		
Slaughter body weight (g)	1653.2 ^b^	1468.4 ^a^	1604.4 ^a,b^	1523.0 ^a,b^	1564.9 ^a,b^	35.53	0.009
HCW (g)	1276.5 ^c^	1120.6 ^a^	1243.6 ^b,c^	1177.9 ^a,b^	1181.4 ^a,b^	18.38	0.0001
CCW (g)	1227.4 ^c^	1075.8 ^a^	1210.0 ^b,c^	1133.2 ^a^	1141.1 ^a,b^	18.53	0.0001
Dressing percentage	77.2	76.5	77.7	77.7	75.6	1.499	0.840
Liver	2.31	2.22	2.22	2.28	2.23	0.043	0.565
Gizzard	2.30	2.47	2.34	2.33	2.45	0.072	0.381
Heart	0.64	0.69	0.69	0.74	0.69	0.022	0.070
Proventriculus	0.51	0.54	0.55	0.55	0.54	0.017	0.540
Spleen	0.11	0.16	0.12	0.13	0.12	0.013	0.265
Pancreas	0.26	0.25	0.26	0.28	0.24	0.014	0.304
Duodenum	0.66 ^a^	0.84 ^b^	0.83 ^b^	0.82 ^b^	0.80 ^b^	0.027	0.0003
Ileum	1.36 ^a^	1.54 ^a,b^	1.66 ^b^	1.48 ^a,b^	1.53 ^a,b^	0.050	0.004
Jejunum	1.38 ^a^	1.61 ^b^	1.72 ^b^	1.59 ^b^	1.68 ^b^	0.045	0.0001
Large intestine	0.71	0.37	0.38	0.22	0.39	0.112	0.057
Ceaca	0.82^a^	1.14 ^b^	1.40 ^c^	1.04 ^a,b^	1.23 ^b,c^	0.056	0.0001
Lungs	0.65	0.65	0.68	0.73	0.67	0.031	0.380

^1^ Diets: CON = commercial chicken diet without red grape pomace; dGP = commercial chicken diet containing 100 g/kg untreated red grape pomace; dPEG = commercial chicken diet containing 100 g/kg red grape pomace pre-treated with polyethylene glycol (50 g/kg); ENZ = commercial chicken diet containing 100 g/kg red grape pomace pre-treated with Viscozyme^®^-L (1 g/kg); PENZ = commercial chicken diet containing 100 g/kg red grape pomace pre-treated with polyethylene glycol (50 g/kg) and Viscozyme^®^-L (1 g/kg). ^2^ Parameters: HCW = hot carcass weight; CCW = water cold carcass weight; ^3^ SEM: Standard error of the mean; ^a,b,c^ In a row, dietary treatment means with common superscripts do not differ (*p* > 0.05). ^a,b,c^ In a row, dietary treatment means with common superscripts do not differ (*p* > 0.05).

**Table 7:** Effect of grape pomace-containing diets treated with polyethylene glycol and a cellulolytic enzyme mixture on meat quality parameters of broiler chickens.

Parameters	^1^ Diets					^3^ SEM	*p* Value
	CON	ENZ	dGP	dPEG	PENZ		
Cooking loss (%)	19.19	22.72	21.32	22.91	23.31	1.060	0.058
Shear force (N)	4.84	4.40	4.86	5.65	4.89	0.366	0.225
^2^ WHC (%)	7.82 ^a,b^	7.58 ^a,b^	5.22 ^a^	5.65 ^a,b^	8.32 ^b^	0.763	0.020
Temperature (°C)	14.58	16.55	14.83	15.68	15.78	0.482	0.070
Meat pH	6.95	6.76	6.92	6.91	6.82	0.086	0.500
Lightness (L *)	48.59	49.85	49.76	48.52	49.50	0.614	0.393
Redness (a *)	1.47	1.48	1.51	1.49	1.50	0.014	0.293
Yellowness (b *)	13.75	12.23	14.50	13.45	13.81	0.571	0.128
Chroma	13.85	12.30	14.55	13.54	13.87	0.578	0.141
Hue angle	1.28	1.05	0.84	1.12	1.00	0.180	0.554

^1^ Diets: CON = commercial chicken diet without red grape pomace; dGP = commercial chicken diet containing 100 g/kg untreated red grape pomace; dPEG = commercial chicken diet containing 100 g/kg red grape pomace pre-treated with polyethylene glycol (50 g/kg); ENZ = commercial chicken diet containing 100 g/kg red grape pomace pre-treated with Viscozyme^®^-L (1 g/kg); PENZ = commercial chicken diet containing 100 g/kg red grape pomace pre-treated with polyethylene glycol (5 g/kg) and Viscozyme^®^-L (1 g/kg); ^2^ WHC = water-holding capacity. ^3^ SEM: Standard error of the mean; ^a,b^ In a row, dietary treatment means with common superscripts do not differ (*p* > 0.05). ^a,b^ In a row, dietary treatment means with common superscripts do not differ (*p* > 0.05).
